# Impaired type I interferon activity and inflammatory responses in severe COVID-19 patients

**DOI:** 10.1126/science.abc6027

**Published:** 2020-07-13

**Authors:** Jérôme Hadjadj, Nader Yatim, Laura Barnabei, Aurélien Corneau, Jeremy Boussier, Nikaïa Smith, Hélène Péré, Bruno Charbit, Vincent Bondet, Camille Chenevier-Gobeaux, Paul Breillat, Nicolas Carlier, Rémy Gauzit, Caroline Morbieu, Frédéric Pène, Nathalie Marin, Nicolas Roche, Tali-Anne Szwebel, Sarah H. Merkling, Jean-Marc Treluyer, David Veyer, Luc Mouthon, Catherine Blanc, Pierre-Louis Tharaux, Flore Rozenberg, Alain Fischer, Darragh Duffy, Frédéric Rieux-Laucat, Solen Kernéis, Benjamin Terrier

**Affiliations:** 1 Université de Paris, Imagine Institute Laboratory of Immunogenetics of Pediatric Autoimmune Diseases, INSERM UMR 1163, F-75015 Paris, France.; 2 Department of Internal Medicine, National Reference Center for Rare Systemic Autoimmune Diseases, AP-HP, APHP-CUP, Hôpital Cochin, F-75014 Paris, France.; 3 Institut Pasteur, Laboratory of Dendritic Cell Immunobiology, INSERM U1223, Department of Immunology, F-75015 Paris, France.; 4 Sorbonne Université, UMS037, PASS, Plateforme de cytométrie de la Pitié-Salpêtrière CyPS, F-75013 Paris, France.; 5 Université de Paris, INSERM, U970, PARCC, F-75015 Paris, France.; 6 Service de Microbiologie, AP-HP, APHP-CUP, Hôpital Européen Georges Pompidou, F-75015 Paris, France.; 7 Institut Pasteur, Cytometry and Biomarkers UTechS, CRT, F-75015 Paris, France.; 8 Department of Automated Diagnostic Biology, Hôpital Cochin, APHP, APHP-CUP, F-75014 Paris, France.; 9 Department of Pulmonology, Hôpital Cochin, AP-HP, APHP-CUP, F-75014 Paris, France.; 10 Equipe Mobile d’Infectiologie, Hôpital Cochin, AP-HP, APHP-CUP, F-75014 Paris, France.; 11 Université de Paris, Institut Cochin, INSERM U1016, CNRS UMR8104, F-75006 Paris, France.; 12 Service de Médecine Intensive et Réanimation, Hôpital Cochin, AP-HP, APHP-CUP, F-75014 Paris, France.; 13 Institut Pasteur, Insect-Virus Interactions Unit, UMR 2000, CNRS, Paris, France.; 14 Université de Paris, Pharmacologie et Evaluation des Thérapeutiques Chez l’Enfant et la Femme Enceinte EA7323, F-75006 Paris, France.; 15 Recherche Clinique et Pharmacologie, AP-HP, APHP-CUP, Hôpitaux Cochin Necker, F-75014 Paris, France.; 16 Université de Paris and Sorbonne Université, INSERM, Centre de Recherche des Cordeliers, Functional Genomics of Solid Tumors (FunGeST), F-75006 Paris, France.; 17 Service de Virologie, Hôpital Cochin, AP-HP, APHP-CUP, F-75014 Paris, France.; 18 Department of Paediatric Immuno-Haematology and Rheumatology, AP-HP, APHP.CUP, Hôpital Necker, F-75015 Paris, France.; 19 Collège de France, Paris, France.; 20 Université de Paris, INSERM, IAME, F-75006 Paris, France.; 21 Institut Pasteur, Epidemiology and Modelling of Antibiotic Evasion (EMAE), F-75015 Paris, France.

## Abstract

Coronavirus disease 2019 (COVID-19) is characterized by distinct patterns of disease progression that suggest diverse host immune responses. We performed an integrated immune analysis on a cohort of 50 COVID-19 patients with various disease severity. A distinct phenotype was observed in severe and critical patients, consisting of a highly impaired interferon (IFN) type I response (characterized by no IFN-β and low IFN-α production and activity), which was associated with a persistent blood viral load and an exacerbated inflammatory response. Inflammation was partially driven by the transcriptional factor nuclear factor–κB and characterized by increased tumor necrosis factor–α and interleukin-6 production and signaling. These data suggest that type I IFN deficiency in the blood could be a hallmark of severe COVID-19 and provide a rationale for combined therapeutic approaches.

## Interferons interfere with lung repair

Interferons (IFNs) are central to antiviral immunity. Viral recognition elicits IFN production, which in turn triggers the transcription of IFN-stimulated genes (ISGs), which engage in various antiviral functions. Type I IFNs (IFN-α and IFN-β) are widely expressed and can result in immunopathology during viral infections. By contrast, type III IFN (IFN-λ) responses are primarily restricted to mucosal surfaces and are thought to confer antiviral protection without driving damaging proinflammatory responses. Accordingly, IFN-λ has been proposed as a therapeutic in coronavirus disease 2019 (COVID-19) and other such viral respiratory diseases (see the Perspective by Grajales-Reyes and Colonna). Broggi *et al.* report that COVID-19 patient morbidity correlates with the high expression of type I and III IFNs in the lung. Furthermore, IFN-λ secreted by dendritic cells in the lungs of mice exposed to synthetic viral RNA causes damage to the lung epithelium, which increases susceptibility to lethal bacterial superinfections. Similarly, using a mouse model of influenza infection, Major *et al.* found that IFN signaling (especially IFN-λ) hampers lung repair by inducing p53 and inhibiting epithelial proliferation and differentiation. Complicating this picture, Hadjadj *et al.* observed that peripheral blood immune cells from severe and critical COVID-19 patients have diminished type I IFN and enhanced proinflammatory interleukin-6– and tumor necrosis factor-α–fueled responses. This suggests that in contrast to local production, systemic production of IFNs may be beneficial. The results of this trio of studies suggest that the location, timing, and duration of IFN exposure are critical parameters underlying the success or failure of therapeutics for viral respiratory infections.

*Science*, this issue p. 706, p. 712, p. 718; see also p. 626

## Teaser

Interferons can both enhance and abate the severity of respiratory viral infections.

Early clinical descriptions of the first severe acute respiratory syndrome coronavirus 2 (SARS-CoV-2)–caused coronavirus disease 2019 (COVID-19) cases at the end of 2019 rapidly highlighted distinct patterns of disease progression (*1*). Although most patients experience mild to moderate disease, 5 to 10% progress to severe or critical disease, including pneumonia and acute respiratory failure (*2*, *3*). On the basis of data from patients with laboratory-confirmed COVID-19 from mainland China, admission to intensive care unit (ICU), invasive mechanical ventilation, or death occurred in 6.1% of cases (*1*), and the death rate from recent current French data was 0.70% (*3*). This proportion of critical cases is higher than that estimated for seasonal influenza (*4*). Additionally, relatively high rates of respiratory failure were reported in young adults (aged 50 years and lower) with previously mild comorbidities (such as hypertension, diabetes mellitus, or overweight) (*5*). Severe cases can occur early in the disease course, but clinical observations typically describe a two-step disease progression, starting with a mild-to-moderate presentation followed by a secondary respiratory worsening 9 to 12 days after the first onset of symptoms (*2*, *6*, *7*). Respiratory deterioration is concomitant with extension of ground-glass lung opacities on chest computed tomography (CT) scans, lymphocytopenia, high prothrombin time, and increased D-dimer levels (*2*). This biphasic evolution marked by a substantial increase of acute phase reactants in the blood suggests a dysregulated inflammatory host response, resulting in an imbalance between pro- and anti-inflammatory mediators. This leads to the subsequent recruitment and accumulation of leukocytes in tissues, causing acute respiratory distress syndrome (ARDS) (*8*). However, little is known about the immunological features and the molecular mechanisms involved in COVID-19 severity.

To test the hypothesis of a virally driven hyperinflammation leading to severe disease, we used an integrative approach based on clinical and biological data, in-depth phenotypical analysis of immune cells, standardized whole-blood transcriptomic analysis, and cytokine measurements on a group of 50 COVID-19 patients with variable severity from mild to critical.

COVID-19 patients (*n* = 50) and healthy controls (*n* = 18) were included. Patients’ characteristics are detailed in the supplementary materials and depicted in table S1 and fig. S1. Patients were analyzed after a median duration of 10 days (interquartile range, 9 to 11 days) after disease onset. On admission, the degree of severity of COVID-19 was categorized as mild to moderate (*n* = 15 patients), severe (*n* = 17 patients), and critical (*n* = 18 patients).

**Fig. 1:**
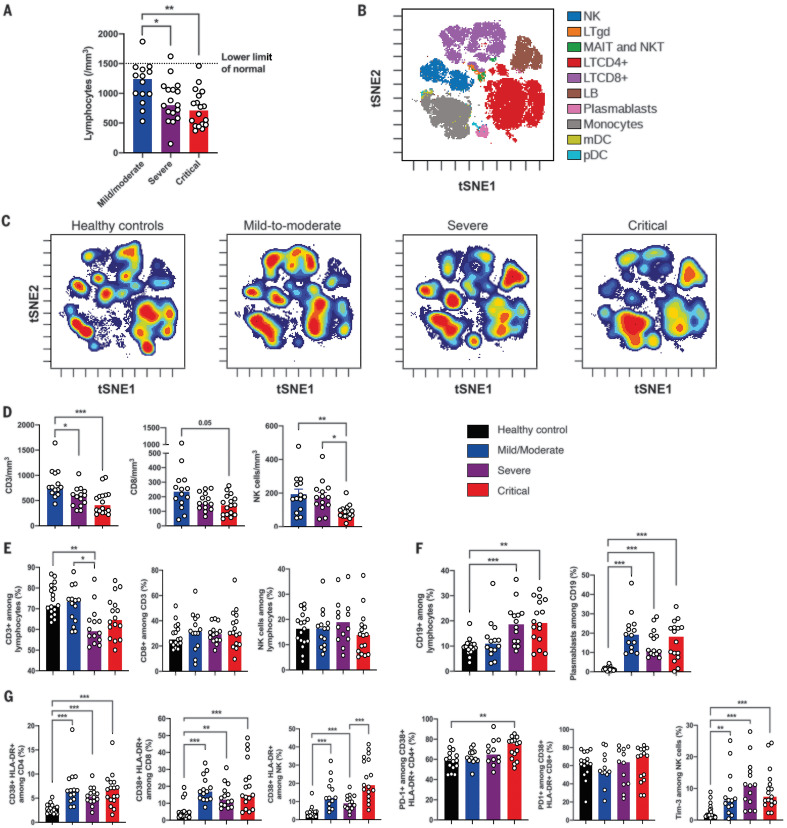
Phenotyping of peripheral blood leukocytes in patients with SARS-CoV-2 infection. (**A**) Lymphocyte counts in whole blood from COVID-19 patients were analyzed between days 8 and 12 after onset of first symptoms, according to disease severity. (**B**) viSNE map of blood leukocytes after exclusion of granulocytes, stained with 30 markers and measured with mass cytometry. Cells are automatically separated into spatially distinct subsets according to the combination of markers that they express. LTgd, γδ T cell; MAIT, mucosal-associated invariant T cell; LB, B lymphocyte. (**C**) viSNE map colored according to cell density across disease severity (classified as healthy controls, mild to moderate, severe, and critical). Red indicates the highest density of cells. (**D**) Absolute number of CD3^+^ T cells, CD8^+^ T cells, and CD3^–^CD56^+^ NK cells in peripheral blood from COVID-19 patients, according to disease severity. (**E** and **F**) Proportions (frequencies) of lymphocyte subsets from COVID-19 patients. (E) Proportions of CD3^+^ T cells among lymphocytes, CD8^+^ T cells among CD3^+^ T cells, and NK cells among lymphocytes. (F) Proportions of CD19^+^ B cells among lymphocytes and CD38hi CD27hi plasmablasts among CD19^+^ B cells. (**G**) Analysis of the functional status of specific T cell subsets and NK cells based on the expression of activation (CD38, HLA-DR) and exhaustion (PD-1, Tim-3) markers. In (D) to (G), data indicate median. Each dot represents a single patient. *P* values were determined with the Kruskal-Wallis test, followed by Dunn’s post-test for multiple group comparisons with median reported; *P < 0.05; **P < 0.01; ***P < 0.001.

As reported in previous studies (*1*, *2*, *8*), lymphocytopenia correlates with disease severity (Fig. 1A). To further characterize this, we used mass cytometry and performed visualization of t-distributed stochastic neighbor embedding (viSNE) (*9*) to compare cell population densities according to disease severity (Fig. 1B). viSNE representation and differentiated cell counts showed a decrease in the density of natural killer (NK) cells and CD3^+^ T cells, including all T cell subsets, that was more pronounced for CD8^+^ T cells. This phenotype was more prominent in severe and critical patients, contrasting with an increase in the density of B cells and monocytes (Fig. 1, C to F). No major imbalance in CD4^+^ and CD8^+^ T cell naïve/memory subsets was observed (fig. S2). Data on T cell polarization and other minor T cell subsets are shown in fig. S3. Plasmablasts were enriched in all infected patients (Fig. 1F), as supported by the increase in genes associated with B cell activation and plasmablast differentiation—such as *IL4R*, *TNFSF13B*, and *XBP1* (fig. S4)—but without any significant increase of serum immunoglobulin concentrations (fig. S5).

We then assessed the functional status of specific T cell subsets and NK cells using markers of activation [CD25, CD38, and human lymphocyte antigen (HLA)–DR] and exhaustion [programmed cell death 1 (PD-1) and Tim-3] (fig. S6A). The CD4^+^ and CD8^+^ T cell populations were characterized by an increase in CD38^+^ HLA-DR^+^–activated T cells in all infected patients, with an expression of PD-1 moderately increasing with disease severity (Fig. 1G and fig. S6B). A similar increase in activated NK cells was found in all infected patients, especially critical patients, and NK cells displayed a significant increase in Tim-3 expression (Fig. 1G). Furthermore, expression of exhaustion-related genes—such as *BATF*, *IRF4*, and *CD274*—significantly increased with disease severity (fig. S6C). High annexin-V expression (by means of flow cytometry) and up-regulation of apoptosis-related genes in the blood from severe and critical patients supported the notion that lymphocytopenia could be partly explained by exacerbated T cell apoptosis (fig. S7).

**Fig. 2:**
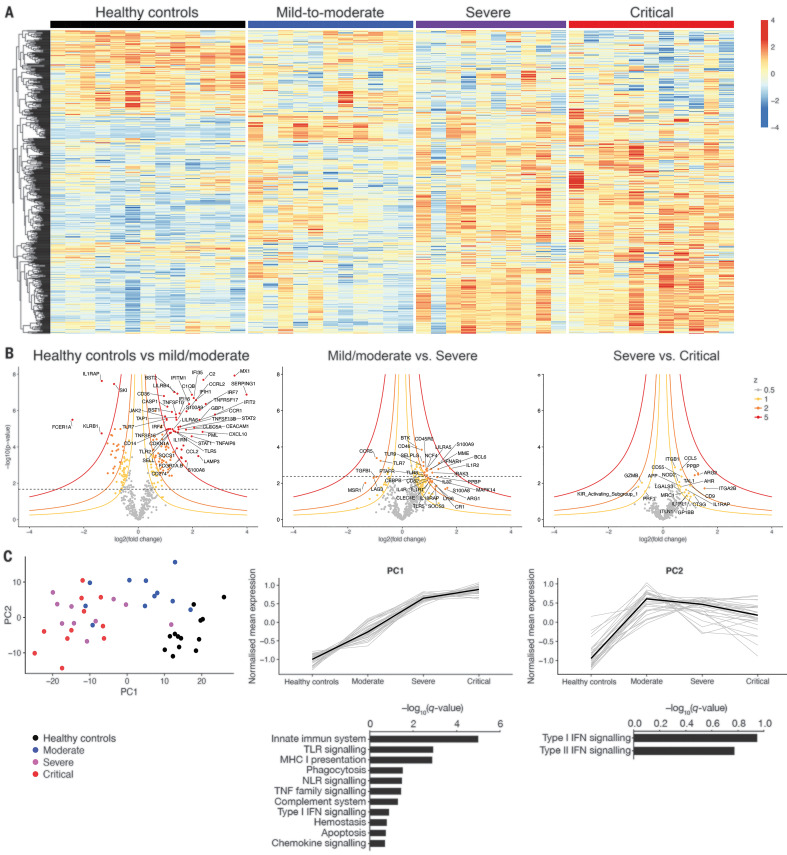
Immunological transcriptional signature of SARS-CoV-2 infection. RNA extracted from patient whole blood and RNA counts of 574 genes were determined by means of direct probe hybridization, using the Nanostring nCounter Human Immunology_v2 kit. (**A**) Heatmap representation of all genes, ordered by hierarchical clustering. Healthy controls (*n* = 13 patients), mild to moderate (*n* = 11 patients), severe (*n* = 10 patients), and critical (*n* = 11 patients). Up-regulated genes are shown in red, and down-regulated genes are shown in blue. (**B**) Volcano plots depicting log10 (*P* value) and log2 (fold change), as well as *z* value for each group comparison (supplementary materials, materials and methods). Gene expression comparisons allowed the identification of significantly differentially expressed genes between severity grades (heathy controls versus mild to moderate, 216 genes; mild to moderate versus severe, 43 genes; severe versus critical, 0 genes). (**C**) (Left) PCA of the transcriptional data. (Middle and right) Kinetic plots showing mean normalized values for each gene and severity grade, where each gray line corresponds to one gene. Median values over genes for each severity grade are plotted in black. Gene set enrichment analysis of pathways enriched in PC1 and PC2 are depicted under corresponding kinetic plot.

To investigate the immunological transcriptional signatures that characterize disease severity, we quantified the expression of immune-related genes in peripheral white blood cells (Fig. 2A). We identified differentially expressed genes as a function of severity grades (Fig. 2B). Unsupervised principal components analysis (PCA) separated patients with high disease severity on principal component 1 (PC1), driven by inflammatory and innate immune response encoding genes (gene set enrichment analysis enrichment score with *q* value < 0.2) (Fig. 2C). PC2, which was enriched in genes encoding proteins involved in both type I and type II interferon (IFN) responses, distinguished mild to moderate patients from the other groups. Collectively, these data suggested a severity grade–dependent increase in activation of innate and inflammatory pathways; by contrast, the IFN response was high in mild to moderate patients, whereas it was reduced in more severe patients.

**Fig. 3:**
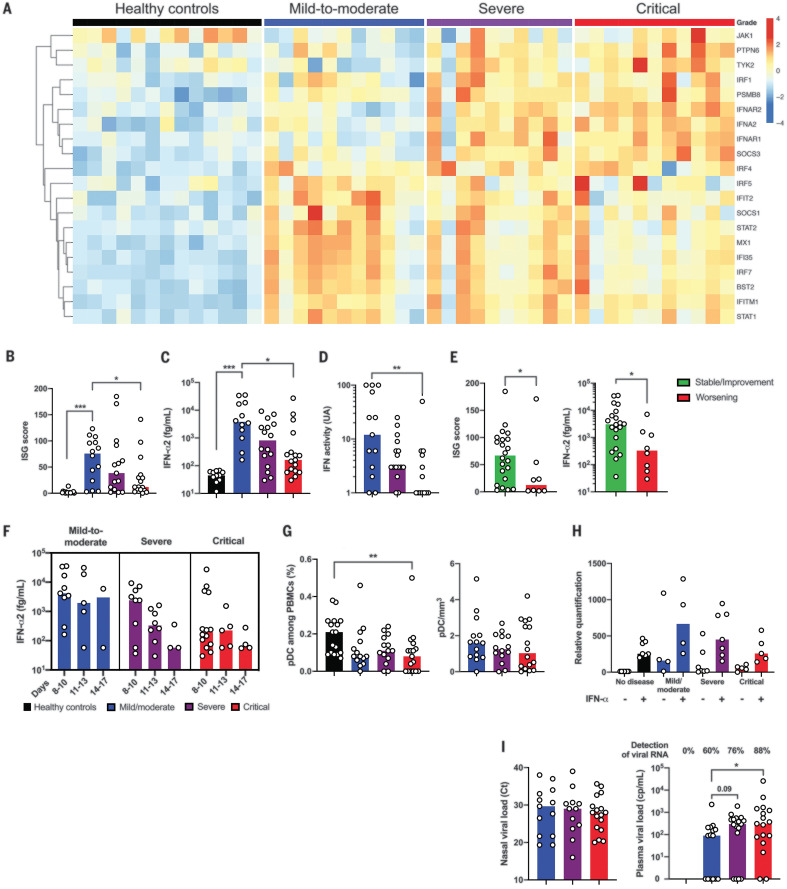
Impaired type I IFN response in patients with severe SARS-CoV-2 infection. (**A**) Heatmap showing expression of type I IFN-related genes by using the reverse transcription- and PCR-free Nanostring nCounter technology in patients with mild-to-moderate (*n* = 11), severe (*n* = 10), and critical (*n* = 11) SARS-CoV2 infection, and healthy controls (*n* = 13). Up-regulated genes are shown in red, and down-regulated genes are shown in blue. (**B**) ISG score based on expression of six genes (*IFI44L*, *IFI27*, *RSAD2*, *SIGLEC1*, *IFIT1*, and *IS15*) measured with quantitative RT-PCR in whole blood cells from mild to moderate (*n* = 14), severe (*n* = 15), and critical (*n* = 17) patients and healthy controls (*n* = 18). (**C**) IFN-α2 (fg/ml) concentration evaluated by use of Simoa and (**D**) IFN activity in plasma according to clinical severity. (**E**) Mild to moderate (*n* = 14) and severe patients (*n* = 16) were separated in two groups depending on the clinical outcome, namely critical worsening requiring mechanical ventilation (to denote severe status). (Left) ISG score and (right) IFN-α2 plasma concentration are shown. (**F**) Time-dependent IFN-α2 concentrations are shown according to severity group. (**G**) Quantification of plasmacytoid dendritic cells (pDCs) as a percentage of PBMCs and as cells/milliliter according to severity group. (**H**) ISG score before and after stimulation of whole blood cells by IFN-α (10^3^UI/ml for 3 hours). (**I**) Viral loads in nasal swabs estimated by means of RT-PCR and expressed in cycle threshold (Ct) and blood viral load evaluated by means of digital PCR. In (B) and (E), ISG score results represent the fold-increased expression compared with the mean of unstimulated controls and are normalized to *GAPDH* (glyceraldehyde phosphate dehydrogenase). In (B) to (I), data indicate median. Each dot represents a single patient. *P* values were determined with the Kruskal-Wallis test, followed by Dunn’s post-test for multiple group comparisons and the Mann-Whitney test for two group comparisons with median reported; *P < 0.05; **P < 0.01; ***P < 0.001.

Type I IFNs are essential for antiviral immunity (*10*). Multiplex gene expression analysis showed an up-regulation of genes involved in type I IFN signaling (such as *IFNAR1*, *JAK1*, and *TYK2*) contrasting with a striking down-regulation of IFN-stimulated genes (ISGs) (such as *MX1*, *IFITM1*, and *IFIT2*) in critical SARS-CoV-2 patients (Fig. 3A). Accordingly, a validated ISG score, based on the mean of expression of six ISGs defining a type I IFN signature (*11*), was significantly reduced in critical patients compared with patients that had mild to moderate infection (Fig. 3B and fig. S8A). *IFN*-β mRNA was undetectable in all infected patients (fig. S8B) as well as IFN-β protein (fig. S8C). Consistent with ISG scores, plasma levels of IFN-α2 protein measured with Simoa digital enzyme-linked immunosorbent assay (ELISA) (*12*) were significantly lower in critical than in mild to moderate patients (Fig. 3C) and correlated with ISG [coefficient of determination (*R*^2^) = 0.30; *P* < 0.0001] (fig. S8D). This result apparently contrasted with the increased detection of *IFNA2* mRNA in most severe patients, albeit at levels just above the limit of detection (fig. S8E). To assess the global type I IFN activity, we used an in vitro cytopathic assay (*13*). IFN activity in serum was significantly lower in severe or critical patients as compared with mild to moderate patients (Fig. 3D). ISG score and plasma levels of IFN-α2 from blood collected before respiratory failure requiring mechanical ventilation revealed that the low type I IFN response preceded clinical deterioration to critical status (Fig. 3E). Furthermore, low plasma levels of IFN-α2 was significantly associated with an increased risk of evolution to critical status [odds ratio (OR) 12; 95% confidence interval (CI) 1.21 to 118; *P* = 0.03]. Analysis in patients for whom multiple time points were available showed distinct patterns of IFN-α production with sustained high response in mild to moderate patients, high but short response in severe patients, and low or no response in critical patients (Fig. 3F). The proportion of plasmacytoid dendritic cells, the main source of IFN-α (*14*), was reduced in infected patients compared with healthy controls, possibly because of migration to sites of infection (*15*), but without any difference between groups (Fig. 3G). We next evaluated the response of whole blood cells to IFN-α stimulation (*11*) and observed a comparable increase in ISG score upon IFN-α stimulation between groups of any severity and controls (Fig. 3H), suggesting that the potential for response to type I IFN was not affected in COVID-19 patients. As a possible consequence of impaired IFN-α production, we used ultrasensitive droplet–based digital polymerase chain reaction (ddPCR) and found an increased plasma viral load in severe and critical patients, which is a possible surrogate marker of uncontrolled lung infection, whereas viral load in nasal swabs by using classical reverse transcription (RT)–PCR was comparable between groups (Fig. 3I). Overall, these data suggest that infected patients had no detectable circulating IFN-β and that an impaired IFN-α production characterized the most severe COVID-19 cases.

**Fig. 4:**
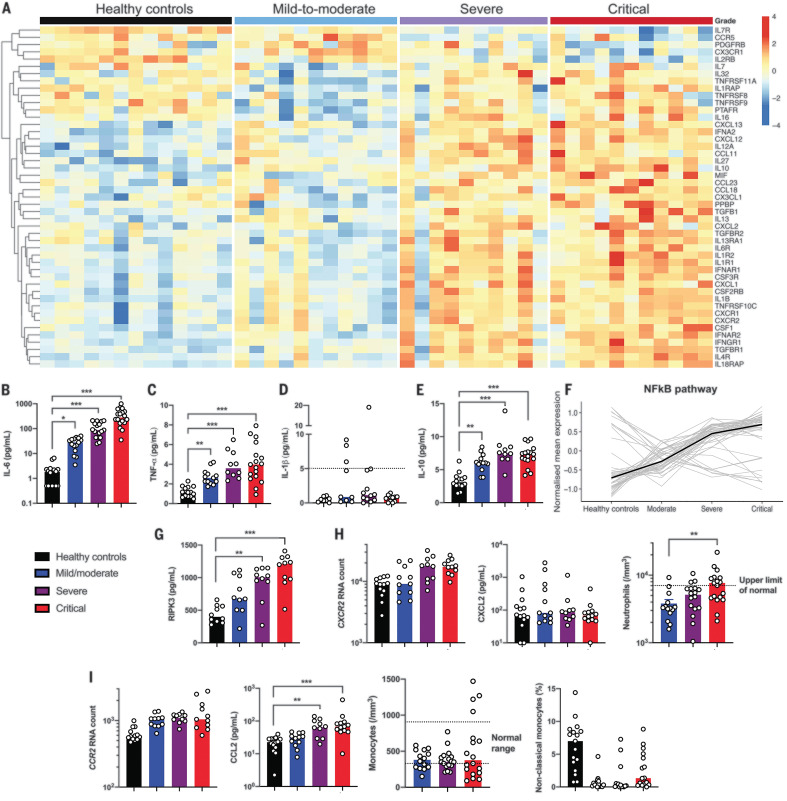
Immune profiling in patients with severe and critical SARS-CoV-2 infection. (**A**) Heatmap showing the expression of cytokines and chemokines that are significantly different in severe and critical patients, ordered by hierarchical clustering. Included are healthy controls (*n* = 13) and mild to moderate (*n* = 11), severe (*n* = 10), and critical (*n* = 11) patients. Up-regulated genes are shown in red, and down-regulated genes are shown in blue. (**B**) IL-6, (**C**) TNF-α, (**D**) IL-1β, and (**E**) IL-10 proteins were quantified in the plasma of patients by using Simoa technology or a clinical-grade ELISA assay (supplementary materials, materials and methods). Each group includes *n* = 10 to 18 patients. The dashed line indicates the limit of detection (LOD). (**F**) Kinetic plots showing mean normalized value for each gene and severity grade. Each gray line corresponds to one gene belonging to the NF-κB pathway. Median values over genes for each severity grade are plotted in black. (**G**) Plasma quantification of RIPK-3. Each group included *n* = 10 patients. (**H**) Absolute RNA count for (left) *CXCR2*, (middle) CXCL2 protein plasma concentration measured with Luminex technology, and (right) blood neutrophil count depending on severity group. The dashed line indicates the upper normal limit. Each group includes *n* = 10 to 13 patients. (**I**) Absolute RNA count for (left) *CCR2*; (middle left) CCL2 protein plasma concentration measured by Luminex technology; and (middle right) blood monocyte count depending on severity group. The dashed lines depict the normal range. (Right) The percentage of nonclassical monocytes, depending on severity grade. Each group shows *n* = 10 to 18 patients. RNA data are extracted from the Nanostring nCounter analysis (supplementary materials, materials and methods). In (B) to (I), data indicate median. Each dot represents a single patient. *P* values were determined with the Kruskal-Wallis test, followed by Dunn’s post-test for multiple group comparisons with median reported; *P < 0.05; **P < 0.01; ***P < 0.001.

Severe COVID-19 was reported to be associated with hypercytokinaemia (*8*, *16*). Cytokine- and chemokine-related genes were found to be increasingly expressed as a function of disease severity in the study cohort (Fig. 4A and fig. S9A). Cytokine whole blood RNA levels did not always correlate with protein plasma levels. Interleukin-6 (IL-6), a key player of the exacerbated inflammatory response in COVID-19 (*17*), was not detected in peripheral blood at the transcriptional level (fig. S9B), contrasting with high amounts of IL-6 protein (Fig. 4B). Expression of IL-6–induced genes—such as *IL6R*, *SOCS3*, and *STAT3*—was significantly increased (fig. S9B), reflecting the activation of the IL-6 signaling pathway. Tumor necrosis factor–α (TNF-α), a key driver of inflammation, was only moderately up-regulated at the transcriptional level (fig. S9C), whereas circulating TNF-α was significantly increased (Fig. 4C). Accordingly, TNF pathway–related genes were also up-regulated, including *TNFSF10* (fig. S9, D and E), which supports TNF-α having an important role in the induction of inflammation. The discrepancy between RNA quantification and protein measurement suggests that cellular sources of TNF-α and IL-6 may be the injured lungs and/or endothelial cells. Conversely, whereas *IL1B* transcripts were significantly up-regulated (fig. S9F), the active form of IL-1β protein was low (Fig. 4D), which suggests that pro–IL-1β was poorly cleaved and secreted but does not exclude a local production in the lung (*15*). Circulating IL-1α also was not detected (fig. S9F). These findings contrasted with the detection of high amounts of circulating IL-1 receptor antagonist (IL-1RA) and up-regulation of *IL1R1* transcripts, indicating an active antagonism of IL-1 in critically ill patients (fig. S9F). We also detected *IL10* transcripts and IL-10 protein in both severe or critical patients (Fig. 4E and fig. S9G). IFN-γ was increased in mild to moderate patients, and at a lesser extent in severe patients, but not in critical patients. By contrast, no increase in IL-17A amounts was detected in all infected patients’ groups (fig. S10).

We next explored the expression of transcription factors that may drive this exacerbated inflammation and found that genes specifically up-regulated in severe or critical patients mainly belonged to the NF-κB pathway (Fig. 4F and fig. S11, A and B). Among several triggering pathways, aberrant NF-κB activation can result from excessive innate immune sensor activation by pathogen-associated molecular patterns (PAMPs) (such as viral RNA) and/or damage-associated molecular patterns (DAMPs) (for example, released by necrotic cells and tissue damage). Lactate dehydrogenase (LDH), a marker of necrosis and cellular injury, correlated with disease severity (fig. S1C), and receptor-interacting protein kinase–3 (RIPK-3), a key kinase involved in programmed necrosis and inflammatory cell death, was also significantly increased in severe or critical patients (Fig. 4G) and correlated with LDH (*R*^2^ = 0.47; *P* < 0.0001).

The exacerbated inflammatory response has been associated with a massive influx of innate immune cells—namely, neutrophils and monocytes—which may aggravate lung injury and precipitate ARDS (*15*). We therefore analyzed expression of chemokines and chemokine receptors involved in the trafficking of innate immune cells (Fig. 4A). Although the neutrophil chemokine CXCL2 was detected in the serum but with no difference between groups, its receptor *CXCR2* was significantly up-regulated in severe and critical patients (Fig. 4H). Consistently, severe disease was accompanied with higher neutrophilia (Fig. 4H). The inflammatory response pattern remained increased even after normalization of transcriptional data with neutrophil counts (fig. S12). Monocyte chemotactic factor chemokine (C-C motif) ligand 2 (CCL2) was increased in the blood of infected patients as well as the transcripts of its receptor *CCR2*; this was associated with low counts of circulating inflammatory monocytes (Fig. 4I), suggesting a role for the CCL2/CCR2 axis in the monocyte chemoattraction into the inflamed lungs. These observations are in accordance with published studies in bronchoalveolar fluids from COVID-19 patients, describing the key role of monocytes (*15*). Overall, these results support a framework in which an ongoing inflammatory cascade, in the setting of impaired type I IFN production and high viral load, may be fueled by both PAMPs and DAMPs.

In this study, we identified an impaired type I IFN response in severe and critical COVID-19 patients, accompanied by high blood viral load and an excessive NF-κB–driven inflammatory response associated with increased TNF-α and IL-6. Innate immune sensors, such as Toll-like receptors (TLRs) and retinoic acid inducible gene I (RIG-I)–like receptors, play a key role in controlling RNA virus by sensing viral replication and alerting the immune system through the expression of a diverse set of antiviral genes (*18*). Type I IFNs—which include IFN-α, -β and -ω—are hence rapidly induced and orchestrate a coordinated antiviral program via the Janus kinase (JAK)–signal transducers and activators of transcription (STAT) signaling pathway and expression of ISGs (*19*). We observed that SARS-CoV-2 infection was characterized by an absence of circulating IFN-β in COVID-19 patients with all disease-severity grades. In addition, most severe COVID-19 patients displayed impaired IFN-α production that was associated with lower viral clearance. This low type I IFN signature was similar to that observed in young children with severe, but not mild, respiratory syncytial virus infection (*20*) but was remarkably different from the transcriptional response induced by other respiratory viruses such as human parainfluenza virus 3 or influenza A virus, both characterized by a stronger type I IFN response in in vitro experiments (*21*). Although our study was not designed for longitudinal analyses, we observed that low IFN-α plasma levels preceded clinical deterioration and transfer to ICU and that distinct patterns of circulating IFN-α characterized each disease grade. Formal longitudinal studies will be necessary to dissect type I IFN dynamics during SARS-CoV-2 infection. It will be important to assess in severe and critical COVID-19 patients whether this reduced type I IFN production is present at the onset of infection, whether the production is delayed, or whether IFN production is exhausted after an initial peak. Recent data confirmed in cellular and animal models that SARS-CoV-2 inhibited type I and III induction (*21*). These results suggest that SARS-CoV-2 has developed efficient mechanisms to shut down host IFN production.

Conversely, on the host side, several hypotheses may be proposed to explain variability in type I IFN responses to infection. Comorbidities are risk factors for severe COVID-19 that could negatively affect IFN production and in contrast exacerbate inflammatory responses (*22*, *23*). Genetic susceptibility can be also suspected because monogenic disorders in children (*24*) or susceptibility variants in adults (*25*), each involving the type I IFN pathway, have been associated with life-threatening influenza infections. Identification of patients with insufficient IFN production but preserved cellular response to type I IFN could define a high-risk population who might benefit from IFN-α or -β treatment. Benefit and risk as well as the best time window for efficacy of IFN administration nevertheless require to be weighed. Alternatively, IFN-λ (type III IFN) could be tested, as recently proposed (*26*), because the receptor is localized on epithelial cells, which may avoid potential adverse effects caused by type I IFN.

Viral replication within the lungs in conjunction with an increased influx of innate immune cells mediates tissue damage and may fuel an auto-amplification inflammatory loop, including targetable production of IL-6 (*27*) and TNF-α (*28*), potentially driven by NF-κB. Our study provides a case for the inhibition of the TNF-α axis; TNF-α is highly expressed in alveolar macrophages, and anti–TNF-α does not block immune responses in animal models of viral infection (*28*).

Our study has some limitations. First, the study was designed as a cross-sectional analysis, although sequential time points were available for some patients. Second, data provided are mainly derived from the blood and do not allow the assessment of immune responses within the lung. In this respect, data from Bost *et al*. describe a reduced type I IFN signature in bronchoalveolar lavage macrophages from severe COVID-19 patients, supporting the validity of our analysis (*29*).

On the basis of our study, we propose that type I IFN deficiency is a hallmark of severe COVID-19 and infer that severe COVID-19 patients might be potentially relieved from the IFN deficiency through IFN administration and from exacerbated inflammation through adapted antiinflammatory therapies that target IL-6 or TNF-α—a hypothesis worth cautious testing.

## Supplementary Materials

science.sciencemag.org/content/369/6504/718/suppl/DC1 Materials and Methods Supplementary Text Figs. S1 to S12 Tables S1 and S2 References (*30*–*33*)

## Appendix

View/request a protocol for this paper from *Bio-protocol*.
